# Surveillance de l'efficacité d'artéméther-Luméfantrine dans le traitement du paludisme simple à *Plasmodium Falciparum* par étude des mutations des gènes Kelch 13 à Bangui, République Centrafricaine

**DOI:** 10.48327/mtsibulletin.n1.2021.82

**Published:** 2021-03-26

**Authors:** W.S. Nambei, U. Biago, O. Balizou, S. Pounguinza, M. Moyen, C. Ndoua, J.C. Gody

**Affiliations:** 1 Département des sciences biomédicales, Faculté des sciences de la santé, Université de Bangui, République centrafricaine; 2 Complexe pédiatrique de Bangui, République centrafricaine; 3 Laboratoire national de biologie clinique et de santé publique; 4 Service national de lutte contre le paludisme

**Keywords:** *Plasmodium falciparum,*, Enfance, Artémether-luméfantrine, Paludisme simple, K13, Hôpital, Bangui, République centrafricaine, Afrique subsaharienne, *Plasmodium falciparum*, Children, Artemether-lumefantrine, Uncomplicated malaria, K13, Bangui, Hospital, Central African Republic, Sub-Saharan Africa

## Abstract

**Objectif:**

Cette étude vise à surveiller l'efficacité d'artéméther-luméfantrine (AL) dans le traitement du paludisme simple chez les enfants à Bangui.

**Matériels et méthodes:**

Il s'agissait d'une étude transversale à visée évaluative allant du 15 février au 7 mars 2017 chez les enfants de 6 mois à 15 ans pris en charge pour accès simple de paludisme au Complexe pédiatrique de Bangui. Les réponses ont été classées en réponse clinique et parasitologique adéquate (RCPA), en échec parasitologique tardif (EPT), en échec clinique tardif (ECT) et en échec parasitologique précoce (ETP) selon le protocole standardisé de l'OMS. Le gène K 13 a été analysé par la technique de PCR-RFLP puis séquencé.

**Résultats:**

Cinquante-cinq enfants âgés de 6 mois à 15 ans atteints du paludisme simple ont été inclus. Après PCR corrigé, le RCPA a été de 97,8% et l'ECT de 2,2% avec AL. Le cas d'échec observé était dû à une nouvelle infection. Aucune mutation K13 n'a été observée. Tous les échantillons avaient l'allèle de type sauvage. La réduction de l'anémie et de la fièvre a été nette.

**Discussion et conclusion:**

Cette étude confirme que l'artéméther-luméfantrine reste efficace et bien tolérée à Bangui. Cependant, tous les allèles sont de type sauvage en RCA contrairement au Rwanda où une mutation R561H est observée. La surveillance par l'étude des marqueurs moléculaires doit être continue pour détecter plus tôt l'apparition de résistance aux dérivés de l'artémisinine.

## Abstract (en)

**Background:**

Drug resistance remains a major challenge for the management of malaria. This study investigated the efficacy of antimalarial combination artemether-lumefantrine (AL) in the treatment of uncomplicated malaria in Bangui, Central African Republic.

**Materials and methods:**

An evaluative cross-sectional study was conducted between the 15th February and the 7th March 2017 in the Complexe Pédiatrique in Bangui among children aged 6 months to 15 years. Clinical outcome was classified according to WHO criteria as adequate clinical and parasitological response (ACPR), late parasitologic failure (LPF), late clinical failure (LCF), and early treatment failure (ETF). The occurrence of mutations in the K 13 gene was studied by PCR-RFLP.

**Results:**

A total of 55 patients were included. After PCR correction, ACPR at D28 was 97.8% and LCF was 2.2%. Treatment failures were due to new infections. No mutations in the K-13 gene associated with artemisinin resistance were identified. All participants had wild type alleles. The decrease of anemia and fever was substantial.

**Discussion and conclusion:**

This study showed that AL remained efficacious and well-tolerated. However, all participants in Central African Republic had wild type alleles unlike contrary in Rwanda where a R561H mutation has been identified. A regular monitoring of efficacy and study of molecular markers of drug resistance to artemisinin is essential.

## Introduction

En République centrafricaine, la transmission du paludisme est de type holoendémique et pérenne. L'incidence globale du paludisme est de 40,6 cas pour 1000 habitants dans la population générale et de 165,3 cas pour 1000 habitants chez les enfants de moins de 5 ans [10]. Les combinaisons thérapeutiques à base d'artémisinine (CTA) utilisées en première ligne sont: artémether-luméfantrine (AL) et artésunate-amodiaque (AQ-AS) et sont introduites depuis 2005 dans le pays [11]. La lutte contre le paludisme repose prioritairement sur le diagnostic précoce et le traitement des épisodes cliniques afin de prévenir l'émergence d'une résistance de *Plasmodium falciparum* surtout aux dérivés de l'artémisinine.

Ainsi, nous nous proposons d'évaluer: la décroissance parasitaire; la fréquence des réponses cliniques parasitologiques adéquates chez les enfants de 6 mois à 15 ans; la tolérance sous traitement depuis la dernière évaluation en 2010 [12]. Cette étude permet également de déterminer les gènes de résistance impliqués chez les patients qui ont subi un échec thérapeutique.

## Matériels et Méthodes

Il s'agissait d'une étude transversale à visée évaluative allant du 15 février au 07 mars 2017 chez les enfants de 6 mois à 15 ans pris en charge pour accès simple de paludisme au Complexe pédiatrique de Bangui.

Le médicament évalué était artémether 20 mg / luméfantrine 120 mg disposant d'une autorisation de mise sur le marché centrafricain (AMM), fabriqué par Ipca Laboratoire Ltd, fourni gracieusement par l'OMS et présenté en combinaison fixe de 2 mg/kg pour l'artémether à J0, J1 et J3 et de 12 mg/kg pour la luméfantrine à J0, J1, J3.

La clairance éthique obtenue auprès de la Faculté des Sciences de la Santé de l'Université de Bangui portait le N° 13/UB/FACSS/CSCVPER/16.

### Critères d'inclusion et d'exclusion des patients

Les critères d'inclusion ont été les suivants: patients âgés de 6 mois à 15 ans, avec infection mono spécifique à *P. falciparum*, avec une parasitémie comprise entre 2000 et 200000 formes asexuées /μl; température axillaire supérieure ou égale à 37°2 C; possibilité de suivi; accès facile au Centre de santé; consentement éclairé des responsables de l'enfant.

Les critères d'exclusion étaient les suivants: présence d'un ou plusieurs signes généraux de danger ou de paludisme grave selon OMS 2003(4); présence d'une infection mixte; présence d'une malnutrition sévère; présence d'un polyparasitisme ou d'une co-infection virale ou bactérienne; allergie; association à une maladie grave.

### Traitement et suivi des patients

La première dose du traitement était administrée aux enfants au niveau du Complexe pédiatrique par le personnel soignant. Les patients étaient observés pendant 30 mn et la dose était répétée en cas de vomissements. La deuxième et la troisième dose étaient administrées à domicile par les responsables en charge de l'enfant. L'adhésion au traitement était vérifiée par le personnel soignant le troisième jour sur présentation de la plaquette et explication par la personne responsable de la façon dont le médicament avait été administré.

L'évaluation des réponses cliniques et biologiques a été faite au niveau du centre de santé à J3, J7, J14, J28. Ces réponses ont été classées en réponse clinique et parasitologique adéquate (RCPA), en échec parasitologique tardif (EPT), en échec clinique tardif (ECT) et en échec parasitologique précoce (ETP) selon le protocole standardisé de l'OMS. L'évaluation de la tolérance et des effets secondaires a été faite en notant les effets indésirables au moment de suivi dans le centre de santé par le personnel soignant. Le traitement de substitution était la quinine sulfate utilisée à dose de 30 mg/kg/j pendant 7 jours.

## Collecte des Échantillons et Analyse de Laboratoire

Du sang capillaire a été prélevé sur une lame et colorée au Giema à 20% pour la goutte épaisse (GE) et le frottis sanguin. Le papier filtre a été utilisé pour prélever des échantillons à J0, Jéchec et J28 pour analyse moléculaire.

La lecture a été faite à l'objectif x100 et les résultats de la parasitémie exprimés selon la formule: nombre de parasites asexués x 8000 / nombre de globules blancs comptés par μl de sang.

Une lame sur trois a fait l'objet d'une deuxième lecture par un second microscopiste puis envoyée à OMS Genève (Swiss Tropical Institute of Tropical Medicine and Health) pour un contrôle de qualité. L'hémoglobine a été dosée à J0 et J28 en utilisant l'appareil électronique Lovibond^®^.

### Extraction de L'ADN et PCR-RLFP

L'ADN parasitaire a été extrait à partir du sang prélevé sur du papier filtre à J0 et Jéchec en utilisant la méthode Chelex telle que décrite par Plowe et al [16]. Le gène *msp2* a été amplifié par 35 cycles de PCR utilisant des amorces obtenues à partir de MR4 (E-U) comme décrit par Cattamachi et al [5]. Les gènes K13 ont été amplifiés par PCR tel que décrit par Durashing et al [6]. Les produits PCR ont été séparés par électrophorèse sur gel d'agarose à 2% en présence de bromure d'éthidium (BE). Les marqueurs de poids moléculaire de 100 pb (New England Biolabs) étaient utilisés comme standard d'ADN. La technique de restriction de fragment polymorphique (RLFP) a été utilisée pour le génotypage des gènes.

La technique utilisant le vecteur NTI advance (TM) software (version 11, Invitrogen, Cergy Pontoise, France) pour identifier le polymorphisme de fragment d'ADN (SNP) a été utilisée pour le séquençage.

### Échantillonnage et analyses statistiques

La taille de l'échantillon a été calculée en prenant en compte: la proportion d'échec clinique anticipée dans la population (P) de 10%, du niveau de confiance de 95% et de la précision (d) de 10% pour le site selon le protocole de l'OMS 2003 [14]. La taille de l'échantillon attendue était de: n = (1 + 0,2) x35 = 42 patients. Les tests statistiques descriptifs ont été utilisées pour calculer les tendances centrales: avec P <0,05 en bilatéral, le test a été considéré comme statistiquement significatif avec un intervalle de confiance de 95%. Une programmation sur fichier Excel fournie par le Global Malaria Program (GMP) de l'OMS a été utilisée pour l'analyse statistique et traitement des données.

## Résultats

### Caractéristiques des patients inclus

Au total, 55 patients ont été inclus. L'âge moyen était de 5,6 ± 3,4 ans; le sex-ratio H/F était de 1,12; le poids moyen était de 19,4 ± 8,5 kg; la température axillaire moyenne était de 38,8 ± 0,5 °C; la parasitémie moyenne était de 6277 parasites/μl de sang. Le taux moyen d'hémoglobine à J0 était de 8,73 ±1,26 (extrêmes de 6 à 11,5 g/dl) (Tableau I).

**Tableau I:** Caractéristiques des patients inclus Characteristics of the patients included

Paramètres	Nb = 55	Fréquence (%)
**Tranche d'âge (année)**		
moins de 5	24	43,67
5 à 15	31	56,36
moyenne d'âge 5,6 ± 3,4		
**Sexe**		
homme	29	52,72
femme	26	47,27
**Poids (kg) à J0**		
poids Moyen 19,4 ±8,5	55	100
**Température (°C) à J0**		
température moyenne 38,8±0,5	55	100
**Parasitémie (µl) à j0**		
moyenne géométrique 6277	55	100
**Taux d'Hb (g/dl) à J0**		
taux moyen en Hb 8,73±1,26 g/dl	55	100

### Réponses cliniques et biologiques au traitement reçu

À J28, avant PCR corrigé, le nombre total de patients par protocole était de 47 et les PDV étaient au nombre de 8 soit 14,5%. De ces 47 patients, 44 étaient RCPA soit 93,6% [IC95%: 82,6-98,7] et 3 étaient en ECT soit 6,4% [IC95%: 1,3-17,5]. Le taux moyen d'hémoglobine était de 12,77 ± 1,12 g/dl.

Après PCR-corrigé, l'incidence cumulée de succès à J28 était à 97,8% [IC95% = 0,858-0,997] et l'incidence cumulée de l'échec à J28 était à 2,2% [IC95% 0,003-0,142] (Tableau II).

**Tableau II:** Réponses cliniques et biologiques au traitement artémether-luméfantrine Clinical and laboratory responses to artemether-luméfantrine treatment

Paramètres de suivi	Nb = 55	%	IC 95%
**Avant PCR-corrigé**			
ETP	0	0	[0-7,5]
ECT	3	6,4	[1,3-17,5]
EPT	0	0	[0-7,5]
RCPA	44	93,6	[82,5-98,7]
**Après PCR-corrigé**			
ETP	0	0	[0-7,9]
ECT	1	2,2	[0,1-11,8]
EPT	0	0	[0-7,9]
RCPA	44	97,8	[88,2–99,9]
nombre total patients per protocole	45	45	
nombre total patients PDV/RET	10	10	
nombre total de patients à J3	48	48	

**Fig. 1:**
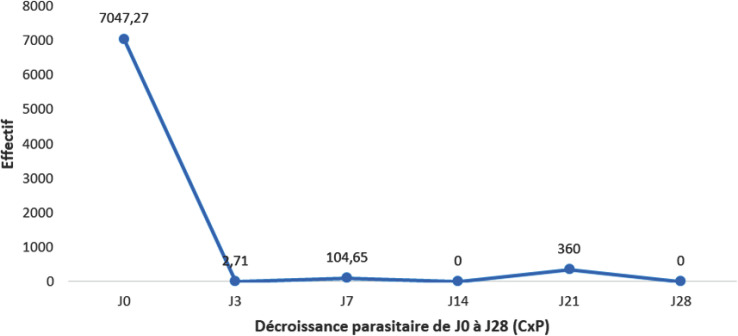
Décroissance parasitaire Parasitemia decrease

La plupart des patients avaient une parasitémie négative à J3. La situation est restée stable à J7, J14, J21 et J28 (Fig. 1).

**Fig. 2:**
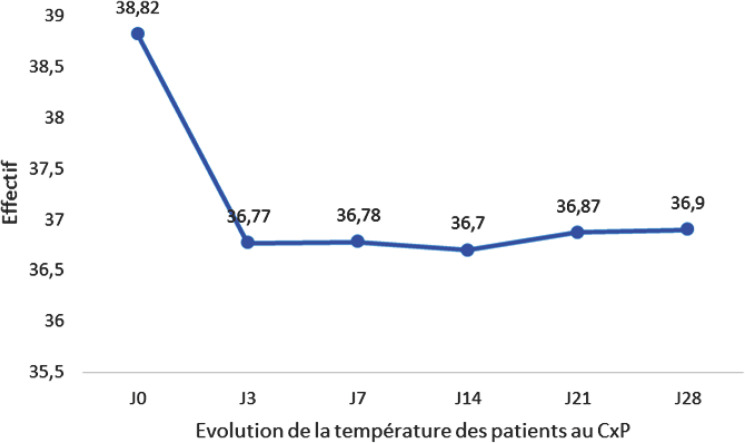
Évolution de la température Temperature evolution

La plupart des patients qui avaient une température élevée à J0 avaient une température normale à J3 et la situation est restée stable à J7, J14, J21et J28 (Fig. 2).

L'incidence des effets secondaires observés au cours de cette étude est faible avec: la fatigue (2%), la douleur abdominale (2%), les nausées (3,4%) et la fièvre (2%).

L'analyse des différents allèles obtenus par PCR pour 2 des 3 échantillons en échec à J21 avec le gène *msp2* (*msp2*_3D7, *msp2*_FC27), plus polymorphe avec une fréquence élevée des allèles obtenus à J0 par rapport au *msp1*(*msp1*_K1, *msp1*_Mad20, *msp1*_RO33) et *glurp* a montré des allèles pour lesquels les paires de base étaient différentes à J0 et J21 (Tableau III).

**Tableau III:** Occurrence des gènes *msp*2 à J0 et J21 Occurrence of msp2 genes on J0 and J21

Code patients	Gènes génotypes	Nb de pb à JO	Nb de pb à J21
CxP_1	*msp2_3D7*	260	230/350
	*msp2_FC27*	480/610	850
CxP_48	*msp2_3D7*	230	210/300/320
	*msp2_FC27*	480/700	850

Pb = paire de base

L'analyse des mutations K1 sur les 55 échantillons analysés n'a montré aucune mutation K 13 au cours de cette étude. Tous les échantillons ont l'allèle de type sauvage. Cependant, le codon A578S observé n'est pas classé comme étant associé à la résistance à l'artémisinine et ses dérivés.

## Discussion

Cette étude confirme l'efficacité clinique et biologique de l'association artémether-luméfantrine utilisée. Après analyse par PCR, nous avons objectivé 97,9% de RCPA. Ces résultats sont en adéquation avec ceux obtenus par certains auteurs au Cameroun [9], Sénégal [7], Gabon [1] et au Nigeria [13]. Ces résultats plaident toujours en faveur de l'utilisation de l'association artémether-luméfantrine du fait de la réduction rapide et significative de la biomasse parasitaire [3]. Elle entraine également la disparition des signes clinique par amélioration de l'anémie, de la fièvre et réduit la transmission des allèles résistants [8]. Toutefois, son coût reste élevé sur le marché national. Ces résultats concordent avec ceux obtenus en RDC en 2014 par Ashley et al [4] et en Centrafrique en 2013 par Nambei et al [12]. La fréquence des effets secondaires était faible au cours de ces travaux. Des résultats similaires ont été obtenus au Sénégal [7] et au Gabon [1]. Ces résultats confirment une bonne tolérance à l'association artémether-luméfantrine utilisée. L'analyse par PCR des échantillons à J0 et Jéchec a permis de conclure que les cas d'échec observés étaient dus probablement à une réinfection et non à une résistance [15]. Par ailleurs, au cours de cette étude, aucune mutation des gènes K13 n'a été observée et tous les allèles sont de type sauvage. Ces mêmes résultats ont été retrouvés par certains auteurs au Sénégal [17], au Gabon [1] et en RDC [15]. Toutefois ces résultats ne suggèrent pas l'absence de la propagation de la résistance aux dérivés de l'artémisinine telle qu'observée en Asie du Sud-Est notamment avec les mutations C580Y, R539T, Y493H et la mutation R561H récemment observée au Rwanda, considérées comme associées in vitro à la résistance ou au ralentissement de la clairance parasitaire [2, 4, 18].

## Conclusion

Cette étude montre que l'association artéméther-luméfantrine reste efficace et bien tolérée à Bangui.

De même aucune mutation K13 n'est observée. Tous les allèles sont de type sauvage. Cependant, la surveillance par l'étude des marqueurs moléculaires doit être continuée pour assurer l'efficacité et ainsi pouvoir détecter plutôt l'apparition de résistance aux dérivés de l'artémisinine.

## Conflits D'intérêts

Les auteurs ne déclarent aucun conflit d'intérêts.

## Remerciements

Les auteurs remercient l'Organisation mondiale de la santé (OMS) pour son appui technique et financier ayant permis la réalisation de ce travail.
